# Early versus delayed enteral nutrition in mechanically ventilated patients with circulatory shock: a nested cohort analysis of an international multicenter, pragmatic clinical trial

**DOI:** 10.1186/s13054-022-04047-4

**Published:** 2022-06-09

**Authors:** Luis Ortiz-Reyes, Jayshil J. Patel, Xuran Jiang, Angel Coz Yataco, Andrew G. Day, Faraaz Shah, James Zelten, Maximiliano Tamae-Kakazu, Todd Rice, Daren K. Heyland

**Affiliations:** 1 Clinical Evaluation Research Unit, Department of Critical Care Medicine, Queen’s University, KGH Research Institute, Kingston Health Sciences Centre, Kingston, ON Canada; 2 Clinical Epidemiology Program, Ottawa Hospital Research Institute, Ottawa, ON Canada; 3 Division of Pulmonary and Critical Care Medicine, Medical College of Wisconsin, Milwaukee, WI USA; 4 Department of Critical Care Medicine and Department of Pulmonary Medicine, Respiratory Institute, Cleveland Clinic, Cleveland, USA; 5 Division of Pulmonary, Allergy and Critical Care Medicine, University of Pittsburgh, Pittsburgh, USA; 6 Pulmonary and Critical Care Medicine, Spectrum Health, Michigan State University, East Lansing, USA; 7 Division of Allergy, Pulmonary, and Critical Care Medicine, Vanderbilt University School of Medicine, Nashville, TN USA

**Keywords:** Enteral nutrition, Vasopressors, EFFORT, ICU, Circulatory shock

## Abstract

**Introduction:**

Real-world evidence on the timing and efficacy of enteral nutrition (EN) practices in intensive care unit (ICU) patients with circulatory shock is limited. We hypothesized early EN (EEN), as compared to delayed EN (DEN), is associated with improved clinical outcomes in mechanically ventilated (MV) patients with circulatory shock.

**Methods:**

We analyzed a dataset from an international, multicenter, pragmatic randomized clinical trial (RCT) evaluating protein dose in ICU patients. Data were collected from ICU admission, and EEN was defined as initiating < 48 h from ICU admission and DEN > 48 h. We identified MV patients in circulatory shock to evaluate the association between the timing of EN initiation and clinical outcomes. The regression analysis model controlled for age, mNUTRIC score, APACHE II score, sepsis, and Site.

**Results:**

We included 626 patients, from 52 ICUs in 14 countries. Median age was 60 years [18–93], 55% had septic shock, 99% received norepinephrine alone, 91% received EN alone, and 50.3% were randomized to a usual protein dose. Forty-two percent of EEN patients had persistent organ dysfunction syndrome plus death at day 28, compared to 53% in the DEN group (*p* = 0.04). EEN was associated with more ICU-free days (9.3 ± 9.2 vs. 5.7 ± 7.9, *p* = 0.0002), more days alive and free of vasopressors (7.1 ± 3.1 vs. 6.3 ± 3.2, *p* = 0.007), and shorter duration of MV among survivors (9.8 ± 10.9 vs. 13.8 ± 14.5, *p* = 0.0002). This trend was no longer observed in the adjusted analysis. There were no differences in ICU/60-day mortality or feeding intolerance rates between groups.

**Conclusion:**

In MV patients with circulatory shock, EEN, as compared to DEN, was associated with improved clinical outcomes, but no longer when adjusting for illness severity. RCTs comparing the efficacy of EEN to DEN in MV patients with circulatory shock are warranted.

**Supplementary Information:**

The online version contains supplementary material available at 10.1186/s13054-022-04047-4.

## Introduction

Up to one-third of patients admitted to an intensive care unit (ICU) are in circulatory shock, which is associated with an increased risk of mortality [1–3]. Death from circulatory shock is often preceded by multiple organ dysfunction syndrome (MODS), which carries a mortality rate of more than 80%. Loss of gut epithelial barrier function (EBF) contributes to gut-derived proinflammatory immune responses that contribute to MODS [4, 5]. Pre-clinical studies have demonstrated early enteral nutrition (EEN) preserves EBF, but there’s a paucity of data in human studies to inform the timing of enteral nutrition (EN) in circulatory shock [6–9]. Furthermore, introducing luminal nutrients in ICU patients may increase the risk of gut-related complications, like non-occlusive mesenteric ischemia and bowel necrosis [10].

Under expert consensus, the Society of Critical Care Medicine (S.C.C.M.) and American Society for Parenteral and Enteral Nutrition (A.S.P.E.N.) 2016 Critical Care Nutrition Guidelines recommend cautiously initiating EN in ICU patients with stable and low vasopressor doses and to withhold EN with escalating vasopressor dose or with enteral feeding intolerance (EFI) [11]. However, no randomized controlled trial (RCT) level evidence informs recommendations for optimal timing of EN in critically ill patients with circulatory shock [12]. Thus, critical care clinicians lack guidance on the optimal timing of EN in critically ill patients with circulatory shock, which may lead to variability in the time to EN initiation.

The purpose of this study is to describe worldwide EN practices in patients with circulatory shock and describe the association between early and delayed enteral nutrition (DEN) on clinical outcomes using a cohort of high nutritional risk critically ill patients enrolled in an ongoing multi-center, multi-national RCT comparing higher to usual protein dose [13]. We hypothesized EEN (< 48 h of ICU admission), as compared to DEN (> 48 h of ICU admission), in mechanically ventilated patients with circulatory shock is associated with improved primary outcome of persistent organ dysfunction plus death (PODS + death) at 28 days.

## Methods

### Study design

We conducted a nested cohort study using data collected prospectively for the ongoing EFFORT Trial (*ClinicalTrials.gov Identifier: NCT03160547*), a multi-center, multi-national, pragmatic, volunteer-driven, registry-based, RCT of nutritionally high-risk critically ill patients randomized to be prescribed a high (≥ 2.2 g/kg/d) or standard protein dose (≤ 1.2 g/kg/d). Full details of the EFFORT Trial are described elsewhere [13]. The study protocol was approved by the Research Ethics Board at Queen’s University. Local institutional review boards (IRBs) at enrolling sites approved the RCT. Participating sites obtained approval for waiver of informed consent; when this was not granted, standard informed consent was obtained.

### Setting

In the main RCT, data were prospectively collected using REDCap [14] between 2018 and 2021 by site investigators from 100 participating ICUs from Argentina, Australia, Brazil, Canada, Greece, Hong Kong, India, Iran, Japan, Mexico, Malaysia, Panama, Puerto Rico, Saudi Arabia, UK, and USA. Data were abstracted from medical records, nursing flow charts, and nutritional assessments in each ICU. In the main trial, all data were collected beginning on ICU Day 1, which was defined as the ICU admission date (not the randomization date). Data were collected from 00:00 h until 23:59 h of the same calendar day. Study and data quality oversight were performed by The Clinical Evaluation Research Unit at Queen’s University, Kingston, ON, Canada (Methods Centre, https://www.ceru.ca). Each registered ICU had an enteral feeding protocol in place and acknowledged having training in critical care nutrition support.

### Participants

We included patients with circulatory shock from the EFFORT trial. For the EFFORT trial, consecutive patients admitted to ICUs were screened and enrolled within the first 96 h of ICU admission if they met the following inclusion criteria: (1) ≥ 18 years old, (2) nutritionally high-risk defined as (minimum one): (2a) Body Mass Index [BMI] < 25/> 35; (2b) moderate or severe malnutrition [as defined by local assessments]; (2c) frailty [clinical frailty scale ≥ 5]; (2d) sarcopenia [SARC-F score ≥ 4]; (2e) expected mechanical ventilation (MV) > 96 h; and (3) requiring MV with actual or expected total duration of MV > 48 h, and (4) eligible to receive nutrition support (EN, Parenteral Nutrition [PN], EN + PN, amino acids only). Exclusion criteria were any of (1) pregnancy, (2) > 96 continuous hours of MV before enrollment, (3) expected death or withdrawal of life-sustaining treatments within 7 days from enrollment, (4) the responsible clinician feels that the patient either needs usual or high protein (lack of equipoise), (5) the patient requires PN only and site does not have products to reach the high protein dose.

For this study, we included patients with (1) circulatory shock, defined as those who received any vasopressors or inotropes (epinephrine, vasopressin, levosimendan, dopamine, phenylephrine, dobutamine, or milrinone) for at least two continuous hours during ICU admission, (2) received EN, and (3) had a complete electronic case report form in REDCap. We excluded patients who received only PN. The data analyzed in this study maintained the same time frame for data collected in the main study (ICU Day 1 = ICU admission date).

### Variables data sources/management

The protein prescription for enrolled patients was determined by the study protocol. Energy calculation and the rest of the clinical assessments/management were at the discretion of the clinical ICU team. Variables for this analysis include baseline demographic data, vasopressor and nutrition data, laboratory data, clinical outcomes, and complications like EFI events.

### Vasopressor therapy

For this analysis, we included daily vasopressor(s)/inotrope(s) infusion (highest hourly rate) if given for at least 2 continuous hours of any dose of norepinephrine (µg/kg/min), epinephrine (µg/kg/min), vasopressin (units/min), dobutamine (µg/kg/min), milrinone (µg/kg/min), levosimendan (µg/kg/min), dopamine (> 5 µg/kg/min), or phenylephrine (> 50 µg/minute). For each vasopressor, a norepinephrine equivalent dose was calculated [15]. The highest hourly vasopressor dose was calculated for the first 48 h treatment period.

### Nutrition therapy variables

The nutrition data were collected starting on ICU Day 1 and until the earlier of the following occurred: (1) 12 ICU days, (2) ICU discharge (alive/death), or (3) until full oral feeds. Data were censored at day 60 in patients discharged alive from ICU to hospital and remained in hospital alive at day 60. Nutrition prescription, daily nutrition data (EN alone, PN + EN, AA), EN formula and protein supplement received, quantity of calories-protein received from all sources, EN interruptions (reasons, duration [time]), quantity of propofol, enteral feeding delivery method, and motility agents received were collected; the EN infusion rate (mL/h) and reasons to withholding EN were not collected in the main study. For this analysis, the nutrition performance included evaluable days (first 12 days in the ICU), as previous described [16–18]. Evaluable days included the day after ICU admission up to the earliest of the day prior to ICU discharge (or death), the first day patients began exclusive permanent oral feeding or 12 days after ICU admission. Nutritional adequacy was defined as the total energy or protein received from all EN or PN sources divided by the baseline prescriptions averaged over the days evaluable for nutrition therapy. The calories from propofol, if administered for more than 6 continuous hours, were included, but calories or proteins from intravenous glucose and amino acid infusions were not included. Early EN was defined, based on guidelines, as EN initiated within 48 h of ICU admission and DEN as initiation after 48 h of ICU admission [11].

### Enteral feeding intolerance, laboratory, and clinical outcome variables

Enteral feeding intolerance (EFI) events were defined and reported by each participating ICU according to their local clinical assessment, and included: high gastric residuals, increased abdominal girth/distention, vomiting/emesis, diarrhea, and subjective discomfort. In the main trial, EFI events were collected only after EN initiation and until it was discontinued. The highest daily creatinine (µmol/L), urea/BUN (mg/dl), triglycerides (mg/dl), and lowest phosphate (mmol/L) values were collected.

### Primary outcome

In mechanically ventilated patients with circulatory shock, we aimed to evaluate the impact of EEN, compared to DEN, on the primary outcome of PODS + death at 28 days. PODS + death is defined as the persistence of organ dysfunction and is present when a critically ill patient is receiving a vasopressor, dialysis, and/or mechanical ventilation and the patient had died at the outcome assessments time point. Time points assessed for PODS + death were Day 14, 28, and 60 post-ICU admission [19]. PODS-free days are defined as the number of days (out of 28 since ICU admission) alive and free of vasopressor, dialysis, and/or mechanical ventilation. Secondary outcomes include ICU length of stay (LOS), hospital LOS, mechanical ventilation LOS, readmission rate, duration of vasopressor use, renal replacement therapy (RRT), ICU and 60-day mortality, and EFI rates. ICU-free days are defined as the number of days; out of 28, an individual is alive and not admitted in an ICU. Vasopressor-free days are defined as the number of days; out of 28, an individual is alive and not requiring vasopressor support.

### Statistical methods

Patient demographic variables, use of vasopressor and inotropic therapies, nutrition practices, laboratory values, and clinical and safety outcomes were compared between those receiving EEN and DEN. Categorical variables were reported as counts and percentages and compared between the EEN and DEN groups by the chi-squared test. Continuous variables were reported as mean and standard deviation (±) (range) and compared between groups by the Mann–Whitney U test. Separate multivariable regression models were estimated to estimate the association between EN timing (EEN vs. DEN) and each of PODS + death at day 28, and 60-day mortality after controlling for age, mNUTRIC score, APACHE II score, and sepsis. Since mNUTRIC and APACHE II scores and binary sepsis status were sometimes missing, we created 100 imputed data sets, performed regression separately on each data set and then combined the results using Rubin’s rules [20]. We first used MCMC with 100 imputations to impute just enough of the missing mNUTRIC and APACHEII values to create a monotonic missing data pattern [21]. We then used monotonic regression to fill in the remaining missing mNUTRIC (linear), APACHEII (linear), and sepsis status (logistic). A subgroup analysis that controlled for sites has been performed to evaluate the effect of EEN on PODS + death at day 28 and 60-day mortality within the subpopulation of enrolled patients. All *p*-values are two-sided without adjustment for multiple tests of significance. A *p*-value less than 0.05 was considered statistically significant. All analyses were conducted using SAS software version 9.4 (SAS Institute Inc., Cary, NC, USA) [22].

## Results

### Participant characteristics

**Fig. 1:**
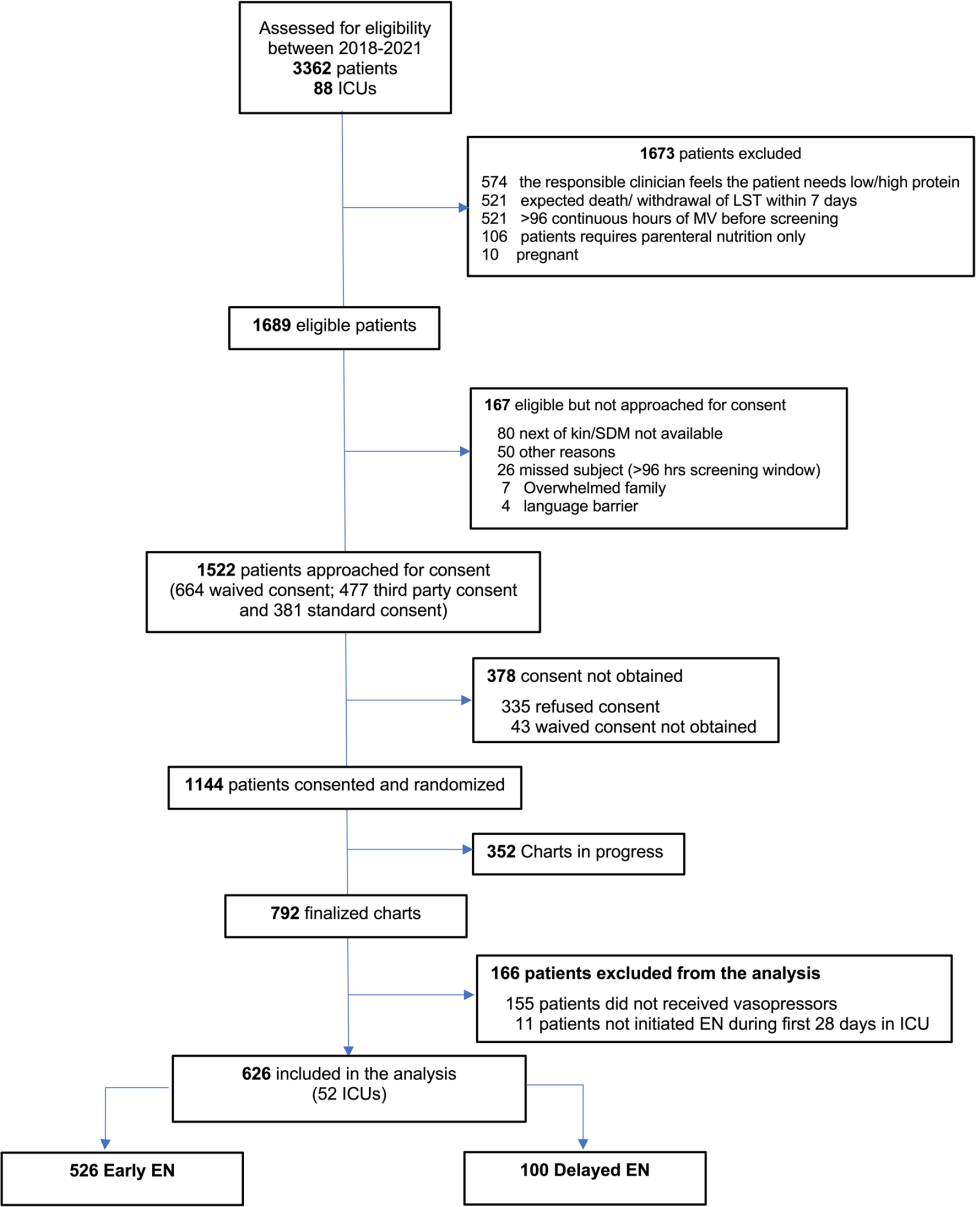
Flow diagram for total ICUs and patients included in the study

A total of 3362 patients in 88 ICUs across 16 countries were screened between January 15, 2018, and September 15, 2021, for inclusion in the main study. Of these, 1144 were randomized. Among 792 finalized charts, 155 patients did not receive vasopressors and 11 did not receive EN, with 626 patients from 52 ICUs in 14 countries included in the analysis; 50.3% (*n* = 315) patients were randomized into the usual protein group. Of these, 526 received EEN and 100 received DEN (Fig. 1).

Of the 626 patients, 56% were male with a mean and standard deviation (±) age of 57.7 ± 17.0 years. Eighty-seven percent of the entire cohort had a medical admission and 55% had septic shock. The EEN group, as compared to the DEN group, had a higher mNUTRIC score (5.0 ± 1.9 vs. 4.3 ± 2.2, *p* = 0.006), a higher APACHE II score (22.9 ± 7.9 vs. 20.9 ± 9.0, *p* = 0.04); and a higher day 1 SOFA score (9.6 ± 3.4 vs. 8.7 ± 4.9, *p* = 0.05), Table 1.

**Table 1:** Baseline demographics and variables

Variable	All (*n* = 626)	Early EN (Within 48 h from ICU admission) (*n* = 526)	Delayed EN (> 48 h from ICU admission) (*n* = 100)	*p* value
*Age (years)* mean ± SD; [range]	57.7 ± 17.0 [18.0–93.0]	58.0 ± 16.9 [18.0–93.0]	56.5 ± 17.4 [20.0–92.0]	0.28
*Gender, n (%)*				0.48
Male	349 (55.8)	290 (55.1)	59 (59.0)	
Female	277 (44.2)	236 (44.9)	41 (41.0)	
*BMI* mean ± SD; [range]	28.2 ± 8.9 [13.8–78.6]	28.3 ± 9.0 [13.8–78.6]	27.6 ± 8.9 [14.9–70.9]	0.28
*mNUTRIC score* mean ± SD; [range]	4.9 ± 2.0 [0.0–9.0]	5.0 ± 1.9 [0.0–9.0]	4.3 ± 2.2 [0.0–9.0]	0.006
≥ 5 *n* (%)	361 (57.7)	322 (61.2)	39 (39.0)	0.01
< 5 *n* (%)	223 (35.6)	182 (34.6)	41 (41.0)	
*Type of admission n (%)*				0.003
Medical	546 (87.2)	465 (88.4)	81 (81.0)	
Surgical Elective	18 (2.9)	10 (1.9)	8 (8.0)	
Surgical Emergency	62 (9.9)	51 (9.7)	11 (11.0)	
*Primary reason/organ system dysfunction for ICU admission, n (%)*				0.44
Cardiovascular/vascular	61 (9.7%)	56 (10.6%)	5 (5.0%)	
Respiratory	273 (43.6%)	231 (43.9%)	42 (42.0%)	
Gastrointestinal	24 (3.8%)	18 (3.4%)	6 (6.0%)	
Neurologic	78 (12.5%)	65 (12.4%)	13 (13.0%)	
Sepsis	109 (17.4%)	87 (16.5%)	22 (22.0%)	
Trauma	45 (7.2%)	38 (7.2%)	7 (7.0%)	
Other	36 (5.8%)	31 (5.9%)	5 (5.0%)	
*Patients with septic shock n (%)*	301 (55.1%)	250 (53.8%)	51 (63.0%)	
Respiratory	184 (33.7%)	157 (33.8%)	27 (33.3%)	0.95
Other sepsis sources *n* (%)	109 (20.0%)	87 (18.7%)	22 (27.2%)	0.69
Central nervous system	8 (1.5%)	6 (1.3%)	2 (2.5%)	0.20
Comorbidities mean ± SD; [range]	2.4 ± 2.1 [0.0–11.0]	2.4 ± 2.1 [0.0–11.0]	2.2 ± 1.9 [0.0–7.0]	0.63
Charleson Comorbidity Index mean ± SD; [range]	1.1 ± 1.6 [0.0–11.0]	1.1 ± 1.6 [0.0–11.0]	1.2 ± 1.6 [0.0–6.0]	0.65
APACHE II Score mean ± SD; [range]	22.6 ± 8.1 [3.0–55.0]	22.9 ± 7.9 [5.0–55.0]	20.9 ± 9.0 [3.0–43.0]	0.04
SOFA Score mean ± SD; [range]	9.4 ± 3.7 [0.0–22.0]	9.6 ± 3.4 [0.0–22.0]	8.7 ± 4.9 [0.0–21.0]	0.05
*Baseline Nutritional Assessment*				
*Nutritionally ‘high-risk’* *n (%)*				0.43
≤ 25	279 (44.6)	229 (43.5)	50 (50.0)	
25– < 35	234 (37.4)	202 (38.4)	32 (32.0)	
≥ 35	113 (18.1)	95 (18.1)	18 (18.0)	
*Moderate to severe malnutrition n (%)*	178 (28.4)	146 (27.8)	32 (32.0)	0.39
*Frailty n (%)*				0.03
≥ *5*	163 (26.0)	129 (24.5)	34 (34.0)	
*1 to 4*	396 (63.3)	342 (65.0)	54 (54.0)	
*Sarcopenia n (%)*				0.08
≥ *4*	90 (14.4%)	70 (13.3%)	20 (20.0%)	
*0–3*	536 (85.6%)	456 (86.7%)	80 (80.0%)	
*From point of screening, projected duration of mechanical ventilation* > *4 days*	495 (79.1%)	411 (78.1%)	84 (84.0%)	
*Participating countries n (%)*				< 0.01
USA	117 (18.7%)	95 (18.1%)	22 (22.0%)	
UK	116 (18.5%)	102 (19.4%)	14 (14.0%)	
Canada	110 (17.6%)	100 (19.0%)	10 (10.0%)	
Mexico	77 (12.3%)	71 (13.5%)	6 (6.0%)	
Argentina	48 (7.7%)	33 (6.3%)	15 (15.0%)	
Brazil	41 (6.5%)	20 (3.8%)	21 (21.0%)	
Panama	23 (3.7%)	23 (4.4%)	0 (0.0%)	
Saudi Arabia	22 (3.5%)	15 (2.9%)	7 (7.0%)	
Greece	20 (3.2%)	19 (3.6%)	1 (1.0%)	
Hong Kong	19 (3.0%)	16 (3.0%)	3 (3.0%)	
Malaysia	16 (2.6%)	15 (2.9%)	1 (1.0%)	
India	8 (1.3%)	8 (1.5%)	0 (0.0%)	
Japan	7 (1.1%)	7 (1.3%)	0 (0.0%)	
Australia	2 (0.3%)	2 (0.4%)	0 (0.0%)	

Numeric variables are represented as “*n*” and percentage (%); mean and standard deviation (±) and [ranges]; body mass index (BMI); Nutrition Risk in Critically ill score is available only for 584 (93.2%) patients; Frailty (Clinical Frailty Scale) score is available only for 559 (89.2%) patients; Frailty (Clinical Frailty Scale); modified Nutritionally ‘high-risk’ (mNUTRIC); Body Mass Index (BMI); Sarcopenia (SARC-F score)

### Descriptive vasopressor and nutrition data

Ninety-nine percent of the entire cohort received norepinephrine (*n* = 621) with an average dose of 0.3 ± 0.6 µg/kg/min, and 90% received 1 vasopressor. The median time, interquartile range [IQR], and range to initiate any vasopressor/inotrope were 2.5 [IQR 0.3–15.0] or (range 0.0–478.0) hours after ICU admission (Table 2). The median time to initiate vasopressor(s) after ICU admission was significantly different between groups (EEN: 2.3 [IQR 0.2–12.0] or (range 0–387.3) vs. DEN: 5.8 [IQR 0.5–45.8] or (range 0–478.0) hours, *p* = 0.002). For the entire cohort, EN was initiated 18.7 [IQR 10.0–36.8] or (range 0.0–434.3) hours after ICU admission. EN alone was delivered to 91% of the entire cohort and 97% had EN delivery into the stomach (gastric feeding tube). We identified 6 feeding practices to initiate EN and ‘EN commenced at a low rate’ was observed in 47% of the cohort, while 2% received trophic feeds, [Additional file 1: Table S1a]. Polymeric EN was the most common formula used (81%), and indirect calorimetry was utilized in 6.9% of the entire cohort. Overall, EN was initiated in a median of 16 [IQR 8–31] or (range 0.3–234.6) hours after vasopressors initiation (EEN: 13.5 [IQR 6.7–21] or (range 0.3–60.8) vs. DEN: 59.4 [IQR 43.2–73.6] or (range 2.3–234.6) hours, *p* < 0.0001).

**Table 2:** Vasopressor and inotropic therapies

	All (*n* = 626)	Early EN (Within 48 h from ICU admission) (*n* = 526)	Delayed EN (> 48 h from ICU admission) (*n* = 100)	*p* value
Time (h) from ICU admission to start vasopressors mean ± SD; median [range]	(626) 20.3 ± 49.5 2.5 [0.0–478.0]	(526) 17.1 ± 43.1 2.3 [0.0–387.3]	(100) 37.0 ± 72.9 5.8 [0.0–478.0]	0.002
Total Duration (days) of vasopressors mean ± SD; median [range]	5.7 ± 3.6 4 [1.0–12.0]	5.4 ± 3.6 4 [1.0–12.0]	6.8 ± 3.8 6 [1.0–12.0]	0.0006
*First 48 h of treatment period*				
Dopamine, µg/kg/min (*n*); mean ± SD; median [range]	(12) 9.3 ± 5.0 7.6 [2.8–18.3]	(10) 8.7 ± 4.4 7.6 [2.8–18.3]	(2) 12.0 ± 9.0 12 [5.7–18.3]	0.75
Dobutamine, µg/kg/min (*n*); mean ± SD; median [range]	(44) 4.6 ± 3.2 4.4 [0.2–17.4]	(38) 4.6 ± 3.2 4.5 [0.2–17.4]	(6) 5.0 ± 3.8 4.2 [2.3–12.5]	0.69
Norepinephrine µg/kg/min (*n*); mean ± SD; median [range]	(621) 0.3 ± 0.6 0.2 [0.0–9.3]	(523) 0.3 ± 0.5 0.2 [0.0–5.2]	(98) 0.3 ± 0.9 0.1 [0.0–9.3]	0.32
Epinephrine, µg/kg/min (*n*); mean ± SD; median [range]	(34) 0.2 ± 0.2 0.1 [0.0–1.0]	(24) 0.2 ± 0.3 0.1 [0.0–1.0]	(10) 0.2 ± 0.1 0.1 [0.0–0.4]	0.81
Phenylephrine, µg/kg/min (*n*); mean ± SD; median [range]	(6) 3.9 ± 3.8 2.2 [0.9–10.5]	(5) 4.5 ± 4.0 3.1 [1.2–10.5]	(1) 0.9 ± .und 0.9 [0.9–0.9]	0.24
Vasopressin, units/min (*n*); mean ± SD; median [range]	(159) 0.0 ± 0.0 0.0 [0.0–0.1]	(139) 0.0 ± 0.0 0.0 [0.0–0.1]	(20) 0.0 ± 0.0 0.0 [0.0–0.1]	0.68
Milrinone, µg/kg/min (*n*); mean ± SD; median [range]	(9) 0.2 ± 0.1 0.2 [0.1–0.3]	(8) 0.2 ± 0.1 0.2 [0.1–0.3]	(1) 0.3 ± .und 0.3 [0.3–0.3]	0.70
Levosimendan, µg/kg/min (*n*); mean ± SD; median [range]	(9) 0.1 ± 0.0 0.1 [0.1–0.2]	(7) 0.1 ± 0.1 0.1 [0.1–0.2]	(2) 0.1 ± 0.0 0.1 [0.1–0.1]	0.36
Vasopressor’s dose at start of EN (*n*); mean ± SD; median [range]	(524) 0.3 ± 0.6 0.2 [0.0–10.0]	(444) 0.3 ± 0.5 0.2 [0.0–6.0]	(80) 0.3 ± 1.1 0.1 [0.0–10.0]	0.07
Norepinephrine-equivalent dose μg/min (*n*); mean ± SD; median [range]	(621) 0.3 ± 0.6 0.2 [0.0–9.3]	(523) 0.3 ± 0.5 0.2 [0.0–5.2]	(98) 0.3 ± 0.9 0.1 [0.0–9.3]	0.32
Proportion of patients with 2 or more vasopressors *n* (%)				0.18
1	564 (90.1%)	475 (90.3%)	89 (89.0%)	
> 2	58 (9.3%)	49 (9.3%)	9 (9.0%)	

Numeric variables are represented as “*n*” and percentage (%); mean and standard deviation ( ±) and [ranges]; µg = microgram; hrs = hours; ICU: intensive care unit

The median time to initiate EN after ICU admission in EEN and DEN groups was 15.5 [IQR 8.4–24.5] or (range 0.0–47.8) versus 67.6 [IQR 57.6–85.3] or (range 48.1–434.3) hours, respectively (*p* < 0.0001). Patients in the EEN group (*n* = 449), as compared to DEN (*n* = 83), during the first 12 ICU days a had greater calorie delivery while on vasopressor (61.5% of prescribed calories or 13.5 kcal/kg/day [range 1.0–31.3] vs. 47.1% of prescribed calories or 10.6 kcal/kg/day [range 0.4–24.8], *p* < 0.001) and greater protein delivery while on vasopressors (53.2% of prescribed protein or 0.8 g/kg/day [range 0.1–2.2] vs. 39.2% of prescribed protein or 0.6 g/kg/day [range 0.0–1.4], *p* < 0.001) [Additional file 1: Table S1a].

### Primary outcome

In the EEN group, 42% (*n* = 220) of patients had PODS + death at 28 days compared with 53% (*n* = 53) in the DEN group (unadjusted odds ratio 0.64 [95% CI 0.42–0.98, *p* = 0.04]). This trend was observed at Day 14 but not Day 60. After controlling for age, mNUTRIC, APACHE II score, and sepsis, the adjusted odds ratio was 0.75 (0.43–1.28, *p* = 0.29), Additional file 2: Table S1b. In addition, EEN was associated with more PODS-free days, as compared to the DEN group (11.0 ± 10.2. vs. 8.5 ± 9.2 days, *p* = 0.03).

### Secondary outcomes and subgroup analysis

The EEN group, as compared to the DEN, had more ICU-free days (out of 28 days) (9.3 ± 9.2 vs. 5.7 ± 7.9 days, *p* = 0.0002), and more vasopressor-free days within 28 days of ICU admission (16.9 ± 9.6 vs. 15.5 ± 9.2 days, *p* = 0.05) (Table 3). In addition, survivors had fewer days of mechanical ventilation (9.8 ± 10.9 vs. 13.8 ± 14.5, *p* = 0.0002), a shorter ICU LOS (13.9 ± 11.1 vs. 20.3 ± 13.6 days, *p* = 0.0001), and a shorter hospital LOS (22.5 ± 13.3 vs. 28.2 ± 15.3 days, *p* = 0.02) with EEN compared to DEN. There were no significant differences in ICU or 60-day mortality rates between groups.

**Table 3:** Overall clinical outcomes

Outcome	All (*n* = 626)	Early EN (Within 48 h from ICU admission) (*n* = 526)	Delayed EN (> 48 h from ICU admission) (*n* = 100)	*p* value
*PODs* + *death at day, n (%)*				
Day 14	329 (52.6)	264 (50.2)	65 (65.0)	0.007
Day 28	273 (43.6)	220 (41.8)	53 (53.0)	0.04
Day 60	256 (40.9)	211 (40.1)	45 (45.0)	0.36
PODs-free days within 28 days mean ± SD; median [range]	10.6 ± 10.1 10 [0.0–26.0]	11.0 ± 10.2 11 [0.0–26.0]	8.5 ± 9.2 5 [0.0–25.0]	0.03
Days alive and free of vasopressors within the first 12 days, mean ± SD; median [range]	7.0 ± 3.1 8 [0.0–12.0]	7.1 ± 3.1 8 [0.0–12.0]	6.3 ± 3.2 6.5 [0.0–12.0]	0.007
Time (days) from ICU admission until discontinuation of vasopressors within the first 28 days mean ± SD; median [range]	6.5 ± 5.6 4 [1.0–28.0]	6.2 ± 5.4 4 [1.0–28.0]	8.1 ± 6.4 6 [1.0–28.0]	0.0008
Alive and vasopressors free days within the first 28 days mean ± SD; median [range]	16.7 ± 9.5 21 [0.0–27.0]	16.9 ± 9.6 21.5 [0.0–27.0]	15.5 ± 9.2 19 [0.0–27.0]	0.05
Ventilator-free days within 28 days from ICU admission mean ± SD; median [range]	11.7 ± 9.8 13 [0.0–28.0]	12.0 ± 9.9 14 [0.0–28.0]	10.2 ± 9.1 9.5 [0.0–28.0]	0.17
Length (days) of mechanical ventilation in survivors (*n*), mean ± SD; median [range]	10.5 ± 11.7 6.1 [0.0–59.5]	9.8 ± 10.9 6.0 [0.0–59.5]	13.8 ± 14.5 8.7 [0.8–57.1]	0.0002
ICU-free days within first 28 days mean ± SD; median [range]	8.7 ± 9.1 5.5 [0.0–26.0]	9.3 ± 9.2 7 [0.0–26.0]	5.7 ± 7.9 0.0 [0.0–24.0]	0.0002
Length of RRT (days) in survivors (*n*), mean ± SD; median [range]	(46) 8.7 ± 8.8 5 [0.0–35.0]	(35) 7.8 ± 7.6 5 [0.0–35.0]	(11) 11.6 ± 11.8 5 [2.0–35.0]	0.57
Length of ICU stay (days) in survivors (*n*), mean ± SD; median [range]	(364) 14.8 ± 11.7 10.7 [1.1–59.3]	(311) 13.9 ± 11.1 10.1 [1.1–59.3]	(53) 20.3 ± 13.6 16.7 [2.7–58.0]	0.0001
Length of hospital stay (days) amongst survivors (*n*), mean ± SD; median [range]	(309) 23.3 ± 13.7 20.3 [1.1–72.7]	(265) 22.5 ± 13.3 20 [1.1–67.2]	(44) 28.2 ± 15.3 22.4 [2.7–72.7]	0.02
*Mortality*				
*at day 14*	126 (20.1%)	109 (20.7%)	17 (17.0%)	0.40
*at day 28*	188 (30.0%)	159 (30.2%)	29 (29.0%)	0.81
ICU mortality *n* (%)	194 (31.0)	161 (30.6)	33 (33.0)	0.55
60-Day mortality *n* (%)	236 (37.7)	200 (38.0)	36 (36.0)	0.81
Readmissions to ICU *n* (%)	27 (4.3)	24 (4.6)	3 (3.0)	0.48
Readmissions to hospital *n* (%)	35 (5.6)	29 (5.5)	6 (6.0)	0.85

*PODS* persistent organ disfunction, *ICU* Intensive Care Unit; Numeric variables are represented as “*n*” and percentage (%); mean and standard deviation ( ±) and [ranges]

**Fig. 2:**
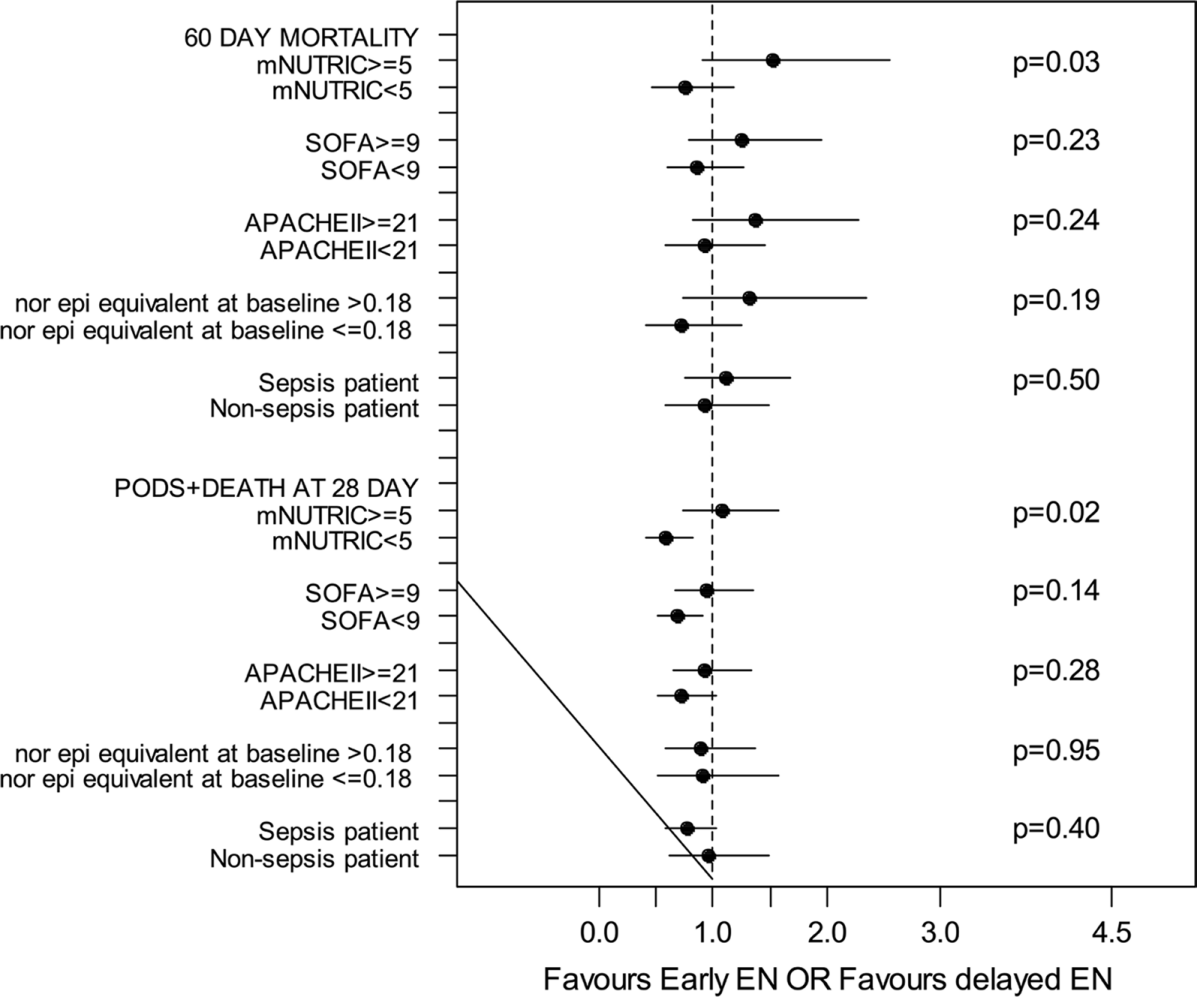
Each horizontal line represents a subgroup analysis for PODS + death at day 28 and 60-day mortality, with means shown by black circles and whiskers representing the 95% confidence interval

There were significant associations between EEN and both 60-day mortality and PODS + death at day 28 in the high and low mNUTRIC groups (Fig. 2). In patients with high mNUTRIC score (≥ 5), EEN was associated with increased risk of 60-day mortality (odds ratio [OR]: 1.52, 95% confidence interval [CI] 0.91–2.54) while there was non-significant association in the opposite direction for patients with low mNUTRIC (test for interaction *p* = 0.03). In patients with a low mNUTRIC score (< 5), EEN was associated with a reduced risk of PODS + death at day 28 (OR: 0.59, CI 0.42–0.83) while there was no association in patients with high mNUTRIC (test for interaction *p* = 0.02).

### Safety outcomes

The rate of enteral feeding intolerance (25% [*n* = 161]) was similar between groups and two events of necrotic bowel/gut ischemia (0.3%) occurred in the EEN group. There were no differences in the serum creatinine, urea, phosphate, and triglycerides serum levels between EEN and DEN groups (Table 4).

**Table 4:** Safety outcomes

Outcomes	All (*n* = 626)	Early EN (Within 48 h from ICU admission) (*n* = 526)	Delayed EN (> 48 h from ICU admission) (*n* = 100)	*p*
*Enteral feeding intolerance, n (%)*				
Overall EFI	161 (25)	–	–	–
High gastric residuals	45 (7.2)	39 (7.4)	6 (6.0)	0.62
Increased abdominal girth or abdominal distention	29 (4.6)	23 (4.4)	6 (6.0)	0.48
Vomiting/emesis	68 (10.9)	55 (10.5)	13 (13.0)	0.45
Diarrhea	15 (2.4)	12 (2.3)	3 (3.0)	0.67
Subjective discomfort	4 (0.6)	4 (0.8)	0 (0.0)	0.38
*Labs from first 12 ICU days*				
Highest creatinine (*n*), mean ± SD; [range] µmol/L	(624) 124.0 ± 113.8 [17.8–1589.2]	(524) 123.6 ± 116.8 [17.8–1589.2]	(100) 125.9 ± 97.2 [29.2–531.5]	0.56
Highest urea/bun (*n*), mean ± SD; [range] mmol/L	(614) 13.3 ± 8.0 [1.8–50.7]	(515) 13.3 ± 8.3 [2.4–50.7]	(99) 13.2 ± 6.8 [1.8–32.8]	0.44
Lowest phosphate (*n*), mean ± SD; [range] mmol/L	(608) 1.1 ± 0.3 [0.5–2.9]	(508) 1.1 ± 0.3 [0.5–2.5]	(100) 1.1 ± 0.4 [0.6–2.9]	0.89
Highest triglycerides (*n*), mean ± SD; [range] mmol/L	(166) 2.3 ± 1.7 [0.3–16.8]	(140) 2.3 ± 1.8 [0.3–16.8]	(26) 2.0 ± 1.1 [0.3–5.5]	0.58

Numeric variables are represented as “*n*” and percentage (%); mean and standard deviation (±) and [ranges]; *EFI* enteral feeding intolerance, *EN* enteral nutrition

## Discussion

In this observational study using worldwide data to describe EN practices in mechanically ventilated patients with circulatory shock, we observed (1) significant variability in EN practices, (2) significant differences in cumulative calorie delivery between EEN and DEN groups during the first 12 ICU days, and (3) the association between EEN and DEN groups and clinical outcomes was no longer significant after adjustment for severity.

Data from this pragmatic study found a significant difference in initiating EN in nutritionally high risk critically ill patients in circulatory shock. The time to initiate EN after ICU admission was a median of 18.7 h but ranged from 0–434.3 h with 16% (*n* = 100) being beyond the recommended 48-h window following ICU admission. In a retrospective propensity matched analysis using a nationwide dataset, Dorken et al. found similar results, with a median time to EN initiation in the late EN group of 79 h [23]. Major nutrition societal guidelines recommend initiating EN in patients with circulatory shock after hemodynamic stability and EN initiation beyond 48 h may be due to greater initial severity of illness [11, 24, 25]. However, our study found the EEN group had greater baseline severity of illness, including higher mNUTRIC, APACHE II, and SOFA scores. The significant delay, beyond the recommended 48 h, may be due to a paucity of data on the optimal timing of EN initiation in circulatory shock. Unlike the recommendation to initiate EEN in the general ICU patient, which is supported by multiple RCTs (that did not include patients with circulatory shock), the evidence base informing timing of EN in circulatory shock consists of pre-clinical and human observational and small RCTs, including a phase II pilot feasibility study demonstrating protocol compliance and safety of early trophic EN in septic shock.^24^ Furthermore, critical care nutrition guidelines recommend, based on expert opinion, to provide tropic EN (low dose) when shock is controlled or that EN be withheld until the patient is fully resuscitated and/or stable [11, 26, 27]. The definition of stability is unspecified and, therefore, ambiguous. The lack of an evidence-based recommendation and, instead, an expert opinion that introduces ambiguity may lead clinicians to delay EN out of fear of complications.

Second, our study found significant differences in calorie (13.5 vs. 10.6 kcal/kg/day, *p* = 0.0005) and protein (0.8 vs. 0.6 g/kg/day, *p* = 0.0001) delivery between EEN and DEN while on vasopressor support, and no differences were observed in the proportion of patients in the DEN and EEN allocated to high and usual protein dose groups in the main RCT. It is unclear if the difference in the quantity of daily calorie and protein represents a clinically important difference. Critically ill patients with circulatory shock who received DEN are more likely to fail to meet their energy and protein requirements, which may expose them to unfavorable clinical outcomes, especially those at high nutritional risk [16, 28–31]. However, in our analysis, patients with lower mNUTRIC score receiving EEN had an increased risk of 60-day mortality and PODS + death at day 28. Our study found EEN group had more days alive and free of vasopressors and surviving patients in the DEN group had a longer length of ICU and hospital LOS. Two patients in the EEN group developed non-occlusive mesenteric ischemia (NOMI)/bowel necrosis (NOBN), and there was no significant difference in EFI rate between EEN and DEN groups. Our findings are discordant with the NUTRIREA-2 trial, which randomized mechanically ventilated patients with predominant septic shock to early EN or early PN [32]. Patients randomized to early EN had a greater EFI rate, including NOMI rate (2 vs. < 1%, *p* = 0.007).^32^ However, the EEN group in NUTRIREA-2 received 17.8 kcal/kg/day while on 0.56 µg/kg/min of norepinephrine [9]. In our study, the EEN group patients received an average of 13.5 kcal/kg/day of calories while on 0.3 µg/kg/min of norepinephrine. These findings align with results from Ohbe et al., which found EEN while on low (< 0.1 µg/kg/min) and medium-dose (0.1–0.3 µg/kg/min) norepinephrine was associated with lower 28-day mortality as compared to DEN with no significant difference in NOMI between EEN and DEN groups (0.2 vs. 0.3%) [31]. The Ohbe et al. study did not account for total calorie intake in reporting NOMI rate. Our findings suggest an EEN dose of up to 13.5 kcal/kg/min may be safe and tolerated while receiving up to 0.3 µg/kg/min of norepinephrine in patients with lower APACHE II and SOFA scores and those with lower nutritional risk (mNUTRIC score < 5).

Third, our study found significant differences in clinical outcomes. The EEN group, as compared to DEN, had a fewer proportion of patients with PODS + death at day 28, shorter ICU and hospital LOS and duration of MV in survivors, and more days alive and free of vasopressors. However, this signal was no longer observed when controlling for severity. The rate of PODS + death was not significant between groups at Day 60, as compared to Day 14 and Day 28, and these data suggest an associated early benefit to EEN which may diminish over time. Our findings align with those from other observational studies. In a small retrospective study, trophic dose EN delivered within 48 h was associated with shorter ICU LOS [33]. More recently, using a national eICU Collaborative Database, Dorken et al. found EEN, as compared to DEN, was associated with shorter ICU LOS [23]. Ours is the first study to find an association between EEN, days alive, and time free of vasopressors. In preclinical and small-scale human studies EEN has been found to preserve EBF [6, 7, 25, 34]. A post hoc analysis of the NUTRIREA-2 trial found the EEN group had higher plasma citrulline levels, a marker of enterocyte mass and function, as compared to the early PN group [9]. These findings suggest EEN may preserve EBF, which may mitigate gut-derived pro-inflammatory immune responses. Clinically, these findings may manifest as more vasopressor free days. In addition, we found significant associations between EEN and both 60-day mortality and PODS + death at day 28 in the high and low mNUTRIC groups. We found EEN, as compared to DEN, in those with a high mNUTRIC score is associated with increased risk of 60-day mortality, as compared to DEN. On the contrary, in patients with a low mNUTRIC score, EEN is associated with less PODS + death, as compared DEN. The sample size of the DEN group was small, which limits the precision of results when comparing mNUTRIC risk groups and the test for interaction. The subgroup effects of mNUTRIC on the outcome of PODS + death at day 28 and 60-day mortality may be real based on key criteria developed to assess credibility of our findings and larger trials are needed to confirm these findings [35].

Our study has several limitations. First, we included mechanically ventilated patients receiving EN and vasoactive agents and cannot account for time intervals between the exposure and outcome of interest. However, we included patients with a minimum of three evaluable days of nutrition to ensure exposure and attempted to capture all time intervals before and after concomitant EN and vasoactive support by defining day 1 as the first ICU admission day, reporting time to initiate EN and time to initiate vasopressor, and including patients who were expected to be mechanically ventilated for > 96 h. Second, we did not explore differences in EN initiation practices based on geography. Nevertheless, despite the presumed heterogeneity of care that might exist across sites around the world, we did observe a significant signal of benefit from early EN. Because of the large sample from diverse real-world settings, our results are maximally generalizable to practices around the world. Third, although we found a statistically significant difference in delivered calories and protein between EEN and DEN groups, we are unsure if these differences represent a meaningful clinically important difference. Fourth, despite controlling for known variables that account for the severity of illness, residual confounding may still be present. Fifth, these results are hypothesis-generating and the findings should be confirmed in randomized controlled trials.

Strengths of our study include reporting real-world practice data from numerous ICUs worldwide that prospectively collected data following a specific protocol, which enhances the validity and generalizability of our findings.

## Conclusions

Our prospective observational study of real-world nutrition practices in mechanically ventilated patients with circulatory shock found significant variability in EN initiation, and EEN, compared to DEN, was associated with improved clinical outcomes, but adjusting for severity reduced this association. RCTs comparing the efficacy of EEN to DEN in mechanically ventilated patients with circulatory shock are warranted.

## Supplementary Information

**Additional file 1. Supplemental Table S1a:** Nutrition practices.

**Additional file 2. Supplemental Table S1b:** Multivariable modeling for Clinical Outcomes.

## Data Availability

The dataset analyzed in the current study is not publicly available but is available from the corresponding author on reasonable request.

## Abbreviations

EN: Enteral nutrition
ICU: Intensive care unit
EEN: Early EN
DEN: Delayed EN
MV: Mechanically ventilated
RCT: Randomized clinical trial
PODS: Persistent organ dysfunction
mNUTRIC: Modified NUTRIC score
NOMI: Non-occlusive mesenteric ischemia
EFI: Enteral feeding intolerance
PN: Parenteral nutrition
NOBN: Non-occlusive bowel necrosis
LOS: Length of stay
OR: Odds ratio
CI: 95% Confidence interval
RRT: Renal replacement therapy
MCMC: Markov chain Monte Carlo method
