# Correction to: Early versus delayed enteral nutrition in mechanically ventilated patients with circulatory shock: a nested cohort analysis of an international multicenter, pragmatic clinical trial

**DOI:** 10.1186/s13054-022-04067-0

**Published:** 2022-06-28

**Authors:** Luis Ortiz‑Reyes, Jayshil J. Patel, Xuran Jiang, Angel Coz Yataco, Andrew G. Day, Faraaz Shah, James Zelten, Maximiliano Tamae‑Kakazu, Todd Rice, Daren K. Heyland

**Affiliations:** 1 Clinical Evaluation Research Unit, Department of Critical Care Medicine, Queen’s University, KGH Research Institute, Kingston Health Sciences Centre, Kingston, ON Canada; 2 Clinical Epidemiology Program, Ottawa Hospital Research Institute, Ottawa, ON Canada; 3 Division of Pulmonary and Critical Care Medicine, Medical College of Wisconsin, Milwaukee, WI USA; 4 Department of Critical Care Medicine and Department of Pulmonary Medicine, Respiratory Institute, Cleveland Clinic, Cleveland, USA; 5 Division of Pulmonary, Allergy and Critical Care Medicine, University of Pittsburgh, Pittsburgh, USA; 6 Pulmonary and Critical Care Medicine, Spectrum Health, Michigan State University, East Lansing, USA; 7 Division of Allergy, Pulmonary, and Critical Care Medicine, Vanderbilt University School of Medicine, Nashville, TN USA

## Correction to: Critical Care (2022) 26:173 10.1186/s13054-022-04047-4

Following publication of the original article [1], the authors identified an error in the co-first authors. The co-first authors indicated wasn’t correct.

The incorrect co-first authors:

^†^Co-first author: Jayshil J. Patel and James Zelten

The correct co-first authors are:

^†^Co-first author: Luis Ortiz‑Reyes and Jayshil J. Patel

The co-first authors have been updated above and the original article [1] has been corrected.
