# Supplementary material for: Early versus delayed enteral nutrition in mechanically ventilated patients with circulatory shock: a nested cohort analysis of an international multicenter, pragmatic clinical trial
Source: Crit Care. 2022 Jun 9;26:173. doi: 10.1186/s13054-022-04047-4 (PMC9185884; doi:10.1186/s13054-022-04047-4)
Supplement: Supplementary file 1 — Additional file 1. Supplemental Table S1a: Nutrition practices. [file 13054_2022_4047_MOESM1_ESM.docx]

|  | | | | |  |
| --- | --- | --- | --- | --- | --- |
| **Table S1a. Nutrition Practices** | | | | |  |
|  | **All**  **(n=626)** | **Early EN**  (Within 48 hrs from ICU admission) **(n=526)** | **Delayed EN**  (>48 hrs from ICU admission)  **(n=100)** | **P value** | |
| **Type of Nutrition** *n (%)* |  |  |  | <0.001 | |
| *EN Only* | 571 (91.2) | 489 (93.0) | 82 (82.0) |  | |
| *EN+PN* | 55 (8.8) | 37 (7.0) | 18 (18.0) |  | |
| **Location of Feeding Tube**  *n (%)* |  |  |  |  | |
| *Gastric* | 607 (97.0) | 510 (97.0) | 97 (97.0) | 0.98 | |
| *Small bowel* | 44 (7.0) | 38 (7.2) | 6 (6.0) | 0.66 | |
| **Timing of Nutrition Support** |  |  |  |  | |
| Time (hrs) to initiate EN from ICU admission  (n); mean ± SD; median [range] | (626) 28.4 ±34.4 18.7 [0.0–434.3] | (526) 17.8 ±12.3 15.5 [0.0–47.8] | (100) 84.2 ±53.8  67.6 [48.1–434.3] | <.0001 | |
| Time (hrs) to initiate EN after vasopressors initiation  (n); mean ± SD; median [range] | (485) 24.3 ±27.3  16 [0.3–234.6] | (396) 15.7 ±11.4  13.5 [0.3–60.8] | (89) 62.6 ±41.3  59.4 [2.3–234.6] | <.0001 | |
| Time (hrs) to initiate EN from vasopressors discontinuation  (n); mean ± SD; median [range] | (25) 25.3 ±31.8  11.5 [2.0–129.6] | (14) 7.8 ±4.9  6.7 [2.0–20.0] | (11) 47.5 ±37.8  35.8 [9.0–129.6] | 0.0001 | |
| Time (hrs) to EN initiation patients receiving a single agent  (n); mean ± SD; median [range] | (424) 26.5 ±28.4 17.4 [0.0–234.9] | (356) 16.7 ±11.9 14.3 [0.0–47.5] | (68) 77.8 ±34.0  67.0 [48.1–234.9] | <.0001 | |
| Time (hrs) to EN initiation patients receiving two or more agents  (n); mean ± SD; median [range] | (198) 30.0 ±34.1  21 [0.0–248.7] | (168) 19.8 ±12.9 17.6 [0.0–47.8] | (30) 87.6 ±53.6  68.9 [48.5–248.7] | <.0001 | |
| **Proportion of patients receiving any type of nutrition while on vasopressors**, n (%) | 593 (94.7) | 505 (96.0) | 88 (88.0) | 0.001 | |
| Duration (hrs) of EN previous vasopressors initiation  (n); mean ± SD; median [range] | (626) 10.4 ±39.4 0.0 [0.0–415.0] | (526) 10.8 ±38.4 0.0 [0.0–363.5] | (100) 8.2 ±44.7  0.0 [0.0–415.0] | 0.01 | |
| NPO (days) previous vasopressors discontinuation  (n); mean ± SD; median [range] | (626) 0.2 ±0.9  0.0 [0.0–9.0] | (526) 0.1 ±0.5  0.0 [0.0–5.0] | (100) 1.0 ±1.8  0.0 [0.0–9.0] | <.0001 | |
| NPO (% of days) previous vasopressors discontinuation  (n); mean ± SD; median [range] | (614) 2.9 ±10.3  0.0 [0.0–100.0] | (515) 1.6 ±7.9  0.0 [0.0–100.0] | (99) 9.2 ±17.0  0.0 [0.0–75.0] | <.0001 | |
| NPO (days) post vasopressors discontinuation  (n); mean ± SD; median [range] | (626) 0.2 ±0.7  0.0 [0.0–5.0] | (526) 0.3 ±0.7  0.0 [0.0–5.0] | (100) 0.1 ±0.4  0.0 [0.0–3.0] | 0.02 | |
| NPO (% of days) post vasopressors discontinuation  (n); mean ± SD; median [range] | (594) 1.0 ±2.8  0.0 [0.0–20.8] | (503) 1.1 ±2.9  0.0 [0.0–20.8] | (91) 0.5±2.1  0.0 [0.0–13.6] | 0.02 | |
| **What was the delivery technique recommended by the physician or dietitian at the initial assessment for EN?** N (%) |  |  |  | 0.004 | |
| *Start at low rate and progress to hourly goal rate* | 295 (47.1) | 259 (49.2) | 36 (36.0) |  | |
| *Trophic feeds: no progression* | 15 (2.4) | 13 (2.5) | 2 (2.0) |  | |
| *Start at hourly goal rate* | 46 (7.3) | 39 (7.4) | 7 (7.0) |  | |
| *Keep Nil Per Os/Nil by Mouth* | 1 (0.2) | 1 (0.2) | 0 (0.0) |  | |
| *Start at or progress to 24 hour volume goal based hourly rate* | 263 (42.0) | 212 (40.3) | 51 (51.0) |  | |
| *Parenteral nutrition* | 6 (1.0) | 2 (0.4) | 4 (4.0) |  | |
| **Nutritional therapy prescription** | n=626 | n=526 | n=100 |  | |
| Was indirect calorimetry used to determine the goal calorie requirement? n(%) |  |  |  | <0.001 | |
| *Yes* | 43 (6.9%) | 27 (5.1%) | 16 (16.0%) |  | |
| *No* | 583 (93.1%) | 499 (94.9%) | 84 (84.0%) |  | |
| energy, kcal/day  mean ± SD; median [range] | 1631.4 ±326.9  1600 [832.0–2964.0] | 1629.2 ±326.2 1600 [832.0–2964.0] | 1643.4 ±331.8  1575.5 [1008.0–2488.0] | 0.82 | |
| energy, kcal/kg/day  mean ± SD; median [range] | 22.9 ±5.0  25 [8.2–41.8] | 22.8 ±5.1  25 [8.7–41.8] | 23.2 ±4.8  25 [8.2–32.7] | 0.27 | |
| protein, grams/day  mean ± SD; median [range] | 115.7 ±39.8  107.3 [42.2–220.0] | 115.3 ±39.8  104 [43.2–220.0] | 117.8 ±40.1  115.3 [42.2–202.0] | 0.54 | |
| protein, grams/kg/day  mean ± SD; median [range] | 1.7 ±0.5  1.3 [0.7–2.5] | 1.7 ±0.5  1.2 [0.7–2.5] | 1.7 ±0.5  2.1 [0.8–2.3] | 0.87 | |
| **Overall nutrition received during the first 12 ICU days** |  |  |  |  | |
| energy received, kcal/day  mean ± SD; median [range] | 1221.6 ±469.1  1223.8 [0.0–2838.5] | 1280.4 ±450.8 1295.4 [74.1–2838.5] | 914.8 ±444.5  948.5 [0.0–1926.5] | <.0001 | |
| protein received, grams/day  mean ± SD; median [range] | 79.3 ±39.9  73.2 [0.0–265.5] | 83.7 ±39.7  75.9 [0.0–265.5] | 56.6 ±32.8  54.8 [0.0–143.6] | 0.001 | |
| adequacy of calories  mean ± SD; median [range] % | 75.7 ±26.9  80.5 [0.0–167.5] | 79.4 ±25.4  83.7 [5.8–167.5] | 56.0 ±26.2  60.7 [0.0–118.7] | <.0001 | |
| adequacy of protein  mean ± SD; median [range] % | 69.0±25.2  74.1 [0.0–177.4] | 72.8 ±23.6  77.5 [0.0–177.4] | 48.8 ±23.1  51.4 [0.0–95.0] | <.0001 | |
| **EN received while on vasopressors during the first 12 ICU days** | n=532 | n=449 | n=83 |  | |
| energy received, kcal/day  mean ± SD; median [range] | 958.5 ±443.9  962.4 [60.0–2170.0] | 993.0 ±440.7  990.7 [60.0–2170.0] | 772.0 ±415.9  725 [60.0–1737.1] | <.0001 | |
| energy received, kcal/kg/day  mean ± SD; median [range] | 13.0 ±6.6  13 [0.4–31.3] | 13.5 ±6.6  13.3 [1.0–31.3] | 10.6 ±6.2  9.6 [0.4–24.8] | 0.0005 | |
| protein, grams/day  mean ± SD; median [range] | 55.7 ±29.3  53.8 [3.0–190.0] | 58.0 ±29.5  54.6 [3.8–190.0] | 42.4 ±24.7  42.2 [3.0–109.0] | <.0001 | |
| protein, grams/kg/day  mean ± SD; median [range] | 0.7 ±0.4  0.7 [0.0–2.2] | 0.8 ±0.4  0.8 [0.1–2.2] | 0.6 ±0.3  0.6 [0.0–1.4] | 0.0001 | |
| Adequacy (%) of calories  mean ± SD; median [range] | 59.3 ±25.4  62.4 [4.5–144.7] | 61.5 ±25.2  65.4 [5.0–144.7] | 47.1 ±23.1  49.1 [4.5–99.3] | <.0001 | |
| Adequacy (%) of protein  mean ± SD; median [range] | 51.0 ±25.0  48.7 [2.6–120.7] | 53.2 ±24.8  51.7 [5.9–120.7] | 39.2 ±22.8  37.5 [2.6–103.5] | <.0001 | |
| Proportions of patients with EN initiated after vasopressor, then vasopressor discontinued and not re–started, n (%) | 25 (4.0) | 14 (2.7) | 11 (11.0) | <0.001 | |
| **Patient receiving any motility agent**  n (%) | 237 (37.9) | 198 (37.6) | 39 (39.0) | 0.80 | |
| **EN interruption** | n=523 | n=453 | n=70 |  | |
| **Total hours**  mean ± SD; median [range] | 21.2 ±18.9  16 [0.6–113.0] | 21.0 ±18.7  16 [0.6–113.0] | 22.8 ±19.9  17.6 [1.0–99.3] | 0.53 | |
| **Interruption reasons,** n (%) |  |  |  |  | |
| Fasting for bedside procedures | 394 (62.9) | 350 (66.5) | 44 (44.0) | <.001 | |
| No enteral access available/enteral access lost, displaced or malfunctioning | 85 (13.6) | 74 (14.1) | 11 (11.0) | 0.41 | |
| Other | 58 (9.3) | 50 (9.5) | 8 (8.0) | 0.63 | |
| Trial of oral intake | 46 (7.3) | 44 (8.4) | 2 (2.0) | 0.03 | |
| NPO because subject palliating | 34 (5.4) | 29 (5.5) | 5 (5.0) | 0.84 | |
| Subject deemed too sick to continue EN | 26 (4.2) | 21 (4.0) | 5 (5.0) | 0.64 | |
| Inotropes, vasopressors requirement | 23 (3.7) | 18 (3.4) | 5 (5.0) | 0.44 | |
| Enteral feeding formula not available | 18 (2.9) | 18 (3.4) | 0 (0.0) | 0.06 | |
| New contraindication to EN | 14 (2.2) | 13 (2.5) | 1 (1.0) | 0.36 | |
| Necrotic bowel/gut ischemia | 2 (0.3) | 2 (0.4) | 0 (0.0) | 0.54 | |
| **Enteral Formulas Types** | n=625 | n=526 | n=99 |  | |
| Arginine enriched formula  mean ± SD % | 5.1 ±22.1 | 5.7 ±23.2 | 2.0 ±14.1 | 0.13 | |
| Fish oil enriched formula  mean ± SD % | 29.1 ±45.5 | 29.5 ±45.6 | 27.3 ±44.8 | 0.66 | |
| Glutamine enriched formula  mean ± SD % | 12.5 ±33.1 | 14.4 ±35.2 | 2.0 ±14.1 | 0.0006 | |
| Elemental formula  mean ± SD % | 33.3 ±47.2 | 34.4 ±47.6 | 27.3 ±44.8 | 0.17 | |
| Polymeric formulas  mean ± SD % | 81.0 ±39.3 | 80.2 ±39.9 | 84.8 ±36.0 | 0.28 | |
| **Main trial allocation groups** |  |  |  | 0.35 | |
| *High Protein Dose* | 311 (49.7%) | 257 (48.9%) | 54 (54.0%) |  | |
| *Usual Protein Dose* | 315 (50.3%) | 269 (51.1%) | 46 (46.0%) |  | |
| Numeric variables are represented as “n” and percentage (%); mean and standard deviation (±) and [ranges]; | | | | |  |
