# Supplementary material for: Early versus delayed enteral nutrition in mechanically ventilated patients with circulatory shock: a nested cohort analysis of an international multicenter, pragmatic clinical trial
Source: Crit Care. 2022 Jun 9;26:173. doi: 10.1186/s13054-022-04047-4 (PMC9185884; doi:10.1186/s13054-022-04047-4)
Supplement: Supplementary file 2 — Additional file 2. Supplemental Table S1b: Multivariable modeling for Clinical Outcomes. [file 13054_2022_4047_MOESM2_ESM.docx]

| **Table S1b – Multivariable modeling for Clinical Outcomes.** | | | | |
| --- | --- | --- | --- | --- |
| **PODs+death at day 28** | **Odds Ratio** | **95% CI** | | **p values** |
| **Early EN** | 0.75 | 0.43 | 1.28 | 0.29 |
| **Age** | 1.02 | 1.00 | 1.03 | 0.03 |
| **NUTRIC score** | 1.05 | 0.88 | 1.25 | 0.57 |
| **Patients with sepsis** | 1.02 | 0.71 | 1.47 | 0.90 |
| **APACHE II score** | 1.00 | 0.97 | 1.04 | 0.98 |
| **60–day mortality** |  |  |  |  |
| Early EN | 1.10 | 0.62 | 1.95 | 0.74 |
| Age | 1.01 | 0.99 | 1.02 | 0.25 |
| NUTRIC score | 1.17 | 0.98 | 1.41 | 0.08 |
| Patients with sepsis | 0.89 | 0.62 | 1.29 | 0.54 |
| APACHEII score | 1.00 | 0.96 | 1.03 | 0.81 |
| **Time to discharge alive from hospital (days)** | **Estimate (SE)** | **p values** | |  |
| Early EN | –0.85 (2.93) | 0.77 | |  |
| Age | 0.16 (0.08) | 0.03 | |  |
| NUTRIC score | 1.40 (0.93) | 0.13 | |  |
| Patients with sepsis | –1.70 (1.96) | 0.38 | |  |
| APACHEII score | –0.08 (0.19) | 0.66 | |  |
